# Economic Evaluations and Equity in the Use of Artificial Intelligence in Imaging Examinations for Medical Diagnosis in People With Dermatological, Neurological, and Pulmonary Diseases: Systematic Review

**DOI:** 10.2196/56240

**Published:** 2025-08-13

**Authors:** Giulia Osório Santana, Rodrigo de Macedo Couto, Rafael Maffei Loureiro, Brunna Carolinne Rocha Silva Furriel, Luis Gustavo Nascimento de Paula, Edna Terezinha Rother, Joselisa Péres Queiroz de Paiva, Lucas Reis Correia

**Affiliations:** 1 PROADI-SUS, Hospital Israelita Albert Einstein, 462 Madre Cabrini Street, Tower A, 5th Floor, São Paulo, SP, 04020-001, Brazil, 55 1197444899; 2 Department de Imagem, Hospital Israelita Albert Einstein, São Paulo, Brazil; 3 Escola de Engenharia de Computação, Universidade Federal de Goiás, Goiânia, Brazil; 4 Grupo de Estudos e Pesquisa em Ciência e Tecnologia (GCITE), Instituto Federal de Goiás, Goiânia, Brazil; 5 Instituto Israelita de Ensino e Pesquisa, Hospital Israelita Albert Einstein, São Paulo, Brazil; 6 Departamento de Medicina Preventiva, Universidade de São Paulo, São Paulo, Brazil

**Keywords:** machine learning, ML, artificial intelligence, AI, algorithm, algorithms, economic evaluation, equity

## Abstract

**Background:**

Health care systems around the world face numerous challenges. Recent advances in artificial intelligence (AI) have offered promising solutions, particularly in diagnostic imaging.

**Objective:**

This systematic review focused on evaluating the economic feasibility of AI in real-world diagnostic imaging scenarios, specifically for dermatological, neurological, and pulmonary diseases. The central question was whether the use of AI in these diagnostic assessments improves economic outcomes and promotes equity in health care systems.

**Methods:**

This systematic review has 2 main components, economic evaluation and equity assessment. We used the PRISMA (Preferred Reporting Items for Systematic Reviews and Meta-Analyses) tool to ensure adherence to best practices in systematic reviews. The protocol was registered with PROSPERO (International Prospective Register of Systematic Reviews), and we followed the PRISMA-E (Preferred Reporting Items for Systematic Reviews and Meta-Analyses - Equity Extension) guidelines for equity. Scientific articles reporting on economic evaluations or equity considerations related to the use of AI-based tools in diagnostic imaging in dermatology, neurology, or pulmonology were included in the study. The search was conducted in the PubMed, Embase, Scopus, and Web of Science databases. Methodological quality was assessed using the following checklists, CHEC (Consensus on Health Economic Criteria) for economic evaluations, EPHPP (Effective Public Health Practice Project) for equity evaluation studies, and Welte for transferability.

**Results:**

The systematic review identified 9 publications within the scope of the research question, with sample sizes ranging from 122 to over 1.3 million participants. The majority of studies addressed economic evaluation (88.9%), with most studies addressing pulmonary diseases (n=6; 66.6%), followed by neurological diseases (n=2; 22.3%), and only 1 (11.1%) study addressing dermatological diseases. These studies had an average quality access of 87.5% on the CHEC checklist. Only 2 studies were found to be transferable to Brazil and other countries with a similar health context. The economic evaluation revealed that 87.5% of studies highlighted the benefits of using AI in dermatology, neurology, and pulmonology, highlighting significant cost-effectiveness outcomes, with the most advantageous being a negative cost-effectiveness ratio of –US $27,580 per QALY (quality-adjusted life year) for melanoma diagnosis, indicating substantial cost savings in this scenario. The only study assessing equity, based on 129,819 radiographic images, identified AI-assisted underdiagnosis, particularly in certain subgroups defined by gender, ethnicity, and socioeconomic status.

**Conclusions:**

This review underscores the importance of transparency in the description of AI tools and the representativeness of population subgroups to mitigate health disparities. As AI is rapidly being integrated into health care, detailed assessments are essential to ensure that benefits reach all patients, regardless of sociodemographic factors.

## Introduction

### Background

Health care systems globally face numerous challenges, including geographical and economic disparities, uneven access to services, and unequal distribution of health care professionals. Patient diversity, influenced by factors such as skin color, ethnicity, and socioeconomic status, adds an extra layer of complexity [1]. However, over the last several years, technological advances in artificial intelligence (AI) have offered promising solutions [2,3]. AI, an interdisciplinary field, aims to develop systems capable of performing tasks that typically require human reasoning [4]. Machine learning, a subfield of AI, creates algorithms capable of learning and improving from experience without explicit programming [5,6]. In health care, AI algorithms have been developed to improve medical image analysis, offering benefits such as automatic recognition of abnormalities and disease prediction, ultimately leading to optimized clinical decision-making, cost reduction, and error prevention [7,8]. Despite these advances, gaps remain in evaluating the economic feasibility and equity implications of AI tools in real-world applications, as highlighted by previous studies [9]. Some critical issues must be addressed to maximize the clinical potential of AI. Methodological limitations, including insufficient validation studies and the lack of transparency in the AI decision-making process, threaten the broader adoption and effectiveness of AI systems in clinical settings [10,11].

Moreover, an emerging issue is bias in the real-world use of AI algorithms, particularly with respect to the populations used to train and validate AI models, potentially leading to skewed results that favor certain ethnic or demographic groups [12]. This bias can result in less effective or misleading results when applied to underrepresented populations, perpetuating existing health care inequalities [13,14]. Voets et al [9] emphasized these disparities, noting the critical need for research that not only evaluates the cost-effectiveness of AI but also addresses how these technologies can reduce inequities in health care access.

Equity in health care advocates for equal access to quality care regardless of personal characteristics such as ethnicity, gender, or socioeconomic status [15]. The inclusion of equity-related data into AI requires careful interpretation to avoid unintended biases [16-19]. Though the relationship between economic evaluation and equity lies in the distributional impact of costs and benefits. By identifying cost-effective strategies, economic evaluations can help allocate resources more equitably, ensuring that underserved populations gain access to innovative diagnostic technologies. For instance, reducing diagnostic costs through AI could enhance access for low-income or remote populations, addressing disparities in health care delivery[18].

Brazil, an upper-middle–income country with many social inequalities, has the largest free public health care system in the world, on which the majority of its population relies [20]. However, socioeconomic barriers create challenges in providing diagnoses, especially in impoverished and remote areas. Digital health initiatives, including AI-powered clinical decision support systems, have proven effective in reducing health disparities and improving access to health services [21]. To tackle these challenges, the Brazilian Ministry of Health’s Support Program for the Institutional Development of the Unified Health System (in Portuguese, “Programa de Apoio ao Desenvolvimento Institucional do Sistema Único de Saúde,” known as PROADI-SUS) supports a collaborative project named “Banco de Imagens” (Bank of Images) to create a nationwide cloud-based repository of medical images, as well as to develop and validate AI algorithms to assist in disease diagnosis.

The selection of dermatological, neurological, and pulmonary diseases was guided by the Brazilian Ministry of Health, which sponsors this study, as these conditions are considered national health priorities. Dermatological diseases, particularly melanoma, were included due to the critical need for early diagnosis and the shortage of specialists. Neurological diseases rely heavily on imaging for diagnosis, where timely intervention is essential; yet, specialist availability remains limited. Pulmonary diseases, particularly tuberculosis (TB), continue to pose significant public health challenges, especially in low-resource settings. The Ministry of Health identified these 3 disease areas as key targets where AI could enhance screening and diagnosis. Given this strategic priority, we conducted a single study encompassing all 3 disease groups.

### Objectives

This systematic review investigates the economic performance and viability of the use of AI in diagnostic imaging examinations in real-world scenarios, particularly for dermatological, neurological, and pulmonary diseases. This review will also assess the potential impact of this intervention on equity in health care systems. The research question guiding this investigation is as follows: “To what extent does the use of AI in imaging examinations for the diagnosis of dermatological, neurological, and pulmonary diseases result in improved economic outcomes, and does it promote equity in health care systems?”

## Methods

### Overview

This study was conducted as a systematic review with two main components. The first component focused on economic evaluations, while the second focused on equity considerations. The PRISMA (Preferred Reporting Items for Systematic Reviews and Meta-Analyses) tool [22] was used to ensure adherence to best practices in systematic reviews (the PRISMA checklist is provided in Checklist 1). A protocol for this systematic review was registered in the PROSPERO (International Prospective Register of Systematic Reviews) database under registration number CRD42023407755. No deviations from the registered protocol occurred during the study. We have published a detailed version of the protocol [23]. In addition, we followed the PRISMA-E (Preferred Reporting Items for Systematic Reviews and Meta-Analyses - Equity Extension) guidelines for systematic reviews with a focus on equity [24]. To structure our research question, we followed the PICO (Population, Intervention, Comparison, and Outcome) framework, as shown in Table 1.

**Table 1.** Population, Intervention, Comparison, and Outcome structure for the systematic review of global economic evaluations and equity studies on the use of artificial intelligence in medical diagnosis in people with dermatological, neurological, and pulmonary diseases.

Acronym	Description	Criteria
P	Population	Patients examined for pulmonary, neurological, and dermatological diseases, considering the entire world population, regardless of income and development of the respective country
I	Intervention	Use of artificial intelligence to support diagnostic decisions in any type of medical imaging
C	Comparison	Conventional or human-based diagnostic methods
O	Outcomes	Full economic evaluation studies, such as cost-effectiveness, cost-utility, cost-benefit, or cost-minimization. We also assessed changes in patient outcomes related to health care access and demographic factors such as age, gender, race, and income, with a focus on equity.

### Eligibility Criteria

Scientific articles reporting on economic evaluations or equity considerations related to the use of AI-based tools in diagnostic imaging. These include (1) dermatology, specifically melanoma and carcinoma; (2) neurology, focusing on conditions with notable radiological findings such as microcephaly, brain atrophy, and hydrocephalus; and (3) pulmonology, focusing on conditions such as TB, lung consolidation, pleural effusion, atelectasis, pneumothorax, mediastinal widening, lung edema, lung opacity, lung lesion, lung cancer, other pleural diseases, and lung nodules. Detailed inclusion and exclusion criteria, including language and year of publication, can be found in Table 2.

**Table 2.** Inclusion and exclusion criteria for the systematic review of global economic evaluations and equity studies on the use of artificial intelligence (AI) in medical diagnosis in people with dermatological, neurological, and pulmonary diseases.

Criteria	Inclusion	Exclusion
Article types	Original articles reporting economic or equity evaluations. Cost-effectiveness, cost-utility, cost-benefit, and cost-minimization analyses were considered. Regarding equity, the authors analyzed reported improvements or declines in patient outcomes based on access to health care and demographic indicators such as age, gender, race, and income.	Articles that were not complete economic evaluations, such as cost analyses, cost description studies, and cost outcome descriptions, were excluded. The exclusion was based on the need to include only studies that provided a complete assessment of both costs and outcomes, allowing for a comprehensive understanding of the economic impact of AI interventions. This approach ensures that the review addresses studies that evaluate both the financial and health-related consequences of AI technologies. Regarding equity, we excluded studies that did not account for diverse samples reflective of the broader population.
Study focus	The use of artificial intelligence-based tools in diagnostic imaging in dermatological, pulmonary, and neurological fields.	Studies focused on other medical areas, such as heart disease, breast cancer, and diabetes. AI clinical validation and studies to predict risks were also excluded.
Language	No restrictions based on language.	No restrictions based on language.
Year of publication	No restrictions based on the year of publication	No restrictions based on the year of publication

### Information Sources

Searches were conducted in the PubMed, Embase, Scopus, and Web of Science databases in March 2023, with no restrictions on publication date. Additional searches were performed to identify the references cited in the retrieved articles.

### Search Strategy

A customized search strategy was defined for each database, using terms related to AI, relevant health conditions or diseases according to eligibility criteria, economic evaluations, and equity. A separate search was conducted for each topic area, as well as a general search without a specific area. The final search terms are provided in the Multimedia Appendices 1-4.

### Data Management

An Excel (Microsoft Corp) spreadsheet was used to manage and organize the records during data extraction and synthesis.

### Selection Process

First, all records retrieved by the search terms in different databases were exported to EndNote software (version X9.3.3; Clarivate), a reference management software. Then, the articles were exported to the Rayyan platform (Qatar Computing Research Institute), which was used to remove duplicates and select articles for screening by reading titles and abstracts.

The selection process consisted of 2 phases. In the first phase, 2 researchers independently assessed and selected articles. Researchers GOS and RML evaluated titles and abstracts from the fields of pulmonology and neurology, while researchers GOS and BCRS evaluated titles and abstracts from the field of dermatology. Researchers GOS and RMC also assessed an additional search related to the use of AI in medical images in general, without a specific field. All disagreements were resolved by a third researcher (LRC).

In the second phase, the selected articles from the first phase underwent full-text review. Researchers GOS and RMC assessed the full texts for all fields and searches. All disagreements were resolved by a third researcher (LRC).

### Data Extraction Process

After selecting the articles, 2 researchers (GOS and RMC) collected the variables of interest for the systematic review from each publication with available data. The following information was prioritized for collection: publication title, year of publication, journal, authors, URL, study type, language, study location, time horizon, corresponding author contact, summary, objectives, methodology, study population, participant demographics, health indicators, access to health care services, equity indicators such as odds ratios and relative risks, study perspective, sample size, statistical significance, applied AI, incremental cost-effectiveness ratio (ICER), cost-benefit or equity of results, quality-adjusted life years (QALY), direction of effect (favorable or not), model applied, sensitivity analysis, initial assessment of methodological quality, conflicts of interest, funding, and transferability of results.

We adopted the World Bank’s 2023 classification of countries by income level [25], which categorizes groups by gross national income per capita calculated using the World Bank’s Atlas method. The groups are low income, US $1135 or less; lower-middle income, US $1136‐$4465; upper-middle income, US $4466‐$13,845; high income, US $13,845 or more.

### Risk of Bias in Individual Studies and Transferability

Three tools were used to assess the methodological quality of the selected studies: (1) the CHEC (Consensus on Health Economic Criteria Checklist) economic evaluation checklist [26]; (i2) the EPHPP (Effective Public Health Practice Project) quality assessment tool for quantitative studies that are not economic evaluations but were selected for their equity results [27]; and (3) the Welte checklist to assess the transferability of technologies used in the selected studies between different countries [26,28]. The methodological quality assessment of the studies was conducted independently by 2 researchers (GOS and RMC). Any discrepancies were resolved through discussion with a third researcher (LGNP).

For the CHEC checklist, points were awarded for each criterion met, so that a maximum of 20 points were available for the evaluation. No points were awarded if the criterion was not fully met. A percentage score was calculated by dividing the number of points awarded by the total number of points on the checklist and multiplying by 100.

For the EPHPP tool, each section was scored 1 (strong) if it met the criteria, 2 (moderate) if at least 1 section was rated as weak, and 3 (weak) if 2 or more sections were rated as weak. Finally, a global score was calculated based on the scores for each section.

For the Welte checklist, points were assigned based on the following criteria: perspective (2 points); discount rate (2 points); medical cost approach (2 points); absolute and relative prices in health care (1 point); practice variation (1 point); incidence and prevalence (1 point); case mix (1 point); life expectancy (1 point); productivity and lost work time (1 point), for a total of 12 points. Studies that scored 10 points or more were considered transferable.

## Results

### Search Results

**Figure 1.**
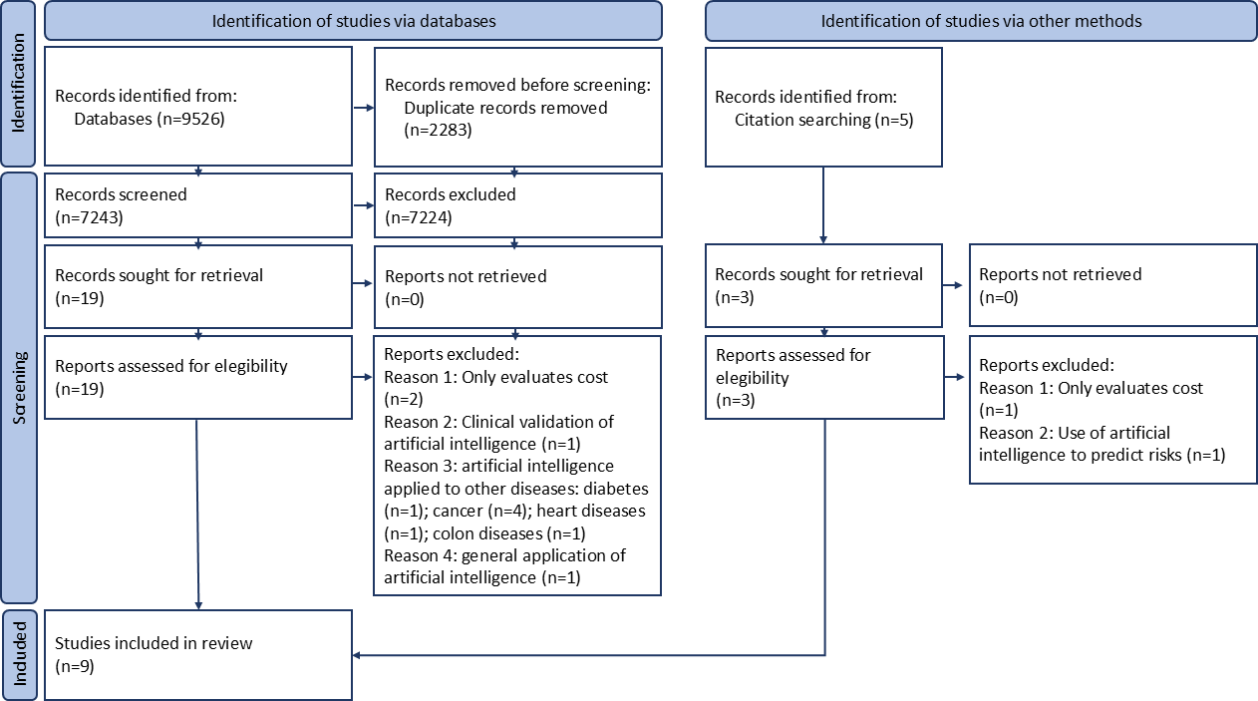
Flowchart illustrating the selection of articles included in the study, based on database searches and additional sources.

The database searches identified a total of 9526 entries, of which 2283 (23.9%) were excluded as duplicates. Of the remaining sample of 7243 articles, 7224 (99.7%) were excluded after screening of titles and abstracts. A total of 19 articles proceeded to the eligibility stage, of which 11 (57.9%) were excluded as they did not meet the inclusion and exclusion criteria. In addition, by reading the references of the selected articles, 3 further articles were identified for full-text reading, of which only 1 (33.3%) was selected for the study. A schematic representation delineating the process of record identification, screening, and exclusion is depicted in Figure 1.

### Overview of the Included Studies

An overview of the included studies is presented in Table 3 and Multimedia Appendix 5. Most of these studies were published in 2021 (n=5, 55.5%), the others in 2022 (n=3, 33.4%) and 2020 (n=1, 11.1%). They focused primarily on pulmonary diseases (n=6, 66.6%; [29-34]), followed by neurological diseases (n=2, 22.3%; [35,36]), and only 1 [37] study (11.1%) focused on dermatological diseases. Of the selected studies, 8 [29-32,34-37] (88.9%) were economic evaluation studies and only 1 (11.1%) [33] addressed equity. The majority of studies (n=5, 55.5%) [29,33-35,37] were conducted in high-income countries, and 3 [30-32] of the 4 (75.0%) studies were conducted in low- to middle-income countries in partnership with institutions located in high-income countries. Notably, 5 [29,31,32,35,37] out of 9 studies (55.5%) reported a conflict of interest. Only 3 [31,32,37] out of 9 studies (33.3%) reported funding, none of which was private.

**Table 3.** General characteristics of the articles selected for the study and the results of the checklists applied for quality assessment.

ID	First author and year of publication	Study type	Place of study	Conflict of interest	Funding
Dermatology, Dentistry, and Ophthalmology					
1	Gomez Rossi et al [37]	Cost-effectiveness analysis	United States, Germany, and Brazil	Yes	Yes, public
Neurology					
2	Leeuwen et al [35]	Cost-effectiveness analysis	United Kingdom	Yes	No
3	Mansour et al [36]	Cost-effectiveness analysis	Egypt	No	Not mentioned
Pulmonology					
4	Adams et al [29]	Cost-minimization analysis	United States	Yes	No
5	Bashir et al [30]	Cost-minimization analysis	Pakistan	No	No
6	MacPherson et al [31]	Cost-effectiveness analysis	Malawi	Yes	Yes, philanthropic institution
7	Nsengiyumva et al [32]	Cost-effectiveness analysis	Pakistan	Yes	Yes, public
8	Seyyed-Kalantari et al [33]	Equity	United States	No	No
9	Ziegelmayer et al [34]	Cost-effectiveness analysis	United States	No	No

### Methodological Quality Assessment

The methodological quality of the studies was assessed using the CHEC checklist, with scores calculated based on the percentage of criteria met (Tables4  5). Scores ranged from 55.0% to 100.0%, with an average of 85.6%.

**Table 4.** Scores for the checklists applied for quality assessment.

ID	CHEC (Consensus Health Economic Criteria) score, (%)	EPHPP (Effective Public Health Practice Project) score	Welte score
1	95%	N/A^a^	10 points (transferable)
2	90%	N/A	10 points (transferable)
3	55%	N/A	6 points (nontransferable)
4	80%	N/A	9 points (nontransferable)
5	75%	N/A	8 points (nontransferable)
6	100%	N/A	7 points (nontransferable)
7	95%	N/A	8 points (nontransferable)
8	N/A	Strong (no weak sections)	N/A
9	95%	N/A	8 points (nontransferable)

a N/A: not applicable

**Table 5.** Results of the checklists applied for quality assessment.

ID	Replaced clinical practice	Description and use of AI^a^ application	Study population	Sample size
1	Diagnosis of melanoma (dermatology) and diabetic retinopathy (ophthalmology) performed solely by clinicians without AI assistance	Convolutional Neural Network (CNN)^b^. Use: decision support for the diagnosis of melanoma, caries, and diabetes	General population (age 50 y), children (age 12 y), people with diabetes (over 40 y), respectively	N/A^c^
2	Detection of intracranial large vessel occlusions in stroke cases performed solely by clinicians without AI support	Not mentioned. Use: computed angiography for stroke detection	People diagnosed with stroke (age 66 y)	71,840
3	Manual ASPECTS assessment for ischemic stroke diagnosis using computed tomography images sent via WhatsApp (Meta)	RAPID ASPECTS by iSchemaView. Use: automated cerebrovascular analysis in computed tomography images for stroke detection	Patients with ischemic stroke who received intravenous thrombolysis (average age 59 y)	122
4	The exclusive use of Lung-RADS classification by radiologists for risk stratification of lung nodules in baseline lung cancer screening	Not mentioned. Use: automated digital chest radiography for lung cancer detection	General population (63% men; average age 61 y; 93% White)	3197
5	The use of radiologists for reading chest x-rays in tuberculosis (TB) screening and triage	CAD4TB (Delft Imaging, Netherlands), Lunit INSIGHT CXR (Lunit, South Korea), qXR (Qure.ai, India), and InferRead DR Chest (Infervision, China). Use: automated digital chest radiography for TB detection	General population (no detailed information)	1.378 million
6	Standard of care tuberculosis (TB) and HIV screening without systematic digital chest x-ray and computer-aided detection (CAD)^e^	CAD4TBv5 Use: automated digital chest radiography for TB detection	Adults (>18 years) with cough symptoms (44% men; average age 33.8 y)	1462
7	Direct microbiological testing (smear microscopy or GeneXpert) for all individuals with presumptive TB, without previous triage	CAD4TBv6 and qXRv2 Use: automated digital chest radiography for TB detection	People with symptoms suggestive of TB (52% men; age 15 y and older, average 33 y)	N/A
8	Traditional radiologist interpretation of chest x-rays for pathology classification	Not mentioned. Use: automated digital chest radiography for pulmonary disease detection	General population (55% men, all ages, 36% [40-60] years, 34% [60-80] years)	129,819
9	Traditional radiologist interpretation of baseline low-dose CT^f^ scans for lung cancer screening	Not mentioned. Use: automated digital chest radiography for lung cancer detection	General population (age 60 y)	N/A

a AI: artificial intelligence.

b Convolutional neural network (CNN), a type of machine learning model designed to analyze visual data, such as images, by automatically identifying patterns and features.

c N/A: not applicable.

d ASPECTS: Alberta stroke program early CT score.

e Computer-aided detection (CAD) refers to the use of computer algorithms to assist in the identification and diagnosis of medical conditions; CAD systems help health care professionals by highlighting potential areas of concern, improving diagnostic accuracy and efficiency.

f CT: computed tomography.

All studies explicitly stated the context of the study and the main question in the introduction. Moreover, they described a study design suitable for their objectives. However, the information on the populations studied was limited. Two studies [30,35] (25.0%) provided no further information, 3 [31,34,37] (37.5%) provided information on the age of participants, 3 [29,32,36] (37.5%) mentioned age and gender, and only 1 study [33] (12.5%) provided information on age, gender, and race or ethnicity. Regarding the use of AI, most studies referred to the technology’s name (n=5, 62.5%). These technologies were mainly used to detect TB (n=3, 37.5%) [30-32], followed by applications in lung cancer [29,34] (n=2, 25.0%), stroke [35,36] (n=2, 25.0%), and 1 study [37] (12.5%) that focused on assisting in melanoma diagnosis.

### Transferability Results

In terms of transferability, only 2 studies [35,37] (25%) were considered transferable to Brazil and other countries with a similar health context. Five studies [29-31,35,36] did not meet the discount rate criterion (62.5%), 1 study [36] did not address medical costs (12.5%), and the majority (75%) [30-32,34,36,37] did not meet the case mix criterion (Tables4  5).

### Equity Assessment

For the equity study, we used the EPHPP checklist and analyzed the study exhibited robust findings, with no sections identified as weak (Tables4  5). The comprehensive selection of databases bolstered the representativeness of the study’s population. The study design was also deemed appropriate for the methodology used. While the study could not analyze race or ethnicity and health insurance confounders for 2 of the 3 databases, it was able to assess gender and age in all cases. The data sources used were appropriately referenced by the authors and were considered reliable. The statistical methods used were deemed appropriate.

### Economic Evaluation

Among the 8 economic evaluation studies identified, the majority (75%, N=6) focused on cost-effectiveness. A cost-effectiveness study evaluates the correlation between the financial resources invested and the benefits obtained, offering an essential analysis for enhancing the economic efficiency of an intervention, project, or program. However, 2 of the studies [29,30] (25.0%) were cost-minimization studies, which focus on identifying the most economical option among alternatives that yield similar outcomes, aiming to minimize expenses while delivering comparable results. Out of the 6 cost-effectiveness studies [31,32,34-37], 2 [34,35] (33.3%) examined the societal perspective, another 2 [31,32] (33.3%) considered the viewpoint of health care providers, and the remaining 2 [36,37] (33.3%) analyzed a combined perspective that encompasses both the health care system and society. Regarding the cost-minimization studies, one [29] adopted the health care system’s perspective, while the other [30] took a combined approach involving both the health care system and society.

The results varied as the studies examined different scenarios with different methods. Nevertheless, the majority of the studies [29,31,32,34-37] (7 out of 8, 87.5%) showed positive results regarding the use of AI in the diagnosis of disease in dermatology, neurology, and pulmonology. The sole study presenting an unfavorable view pertained to the AI’s efficacy in enhancing TB detection [30]. This singular negative outcome may be ascribed to several factors, one of which involves the economic implications of subsequent diagnostic tests required to validate the screening results, which were conducted in 61% of the study’s participants. A summary of the methodological details of these economic evaluation studies is provided in Table 6.

**Table 6.** Details of the economic or equity evaluation of the studies.

ID	Model type	Perspective	Outcome measure	Direction of effect
1	Markov	Patient and health care system	The ICER^a^ for dermatology and ophthalmology was –US $27,580 and –US $38,848 per QALY^b^, respectively. The dominance in favor of AI^c^ depended on small differences in the fee-for-service and assumed postdiagnosis treatment.	Favorable
2	Markov	Society	Over a lifetime horizon, AI resulted in cost savings of US $156 (0.23%) and an incremental gain of 0.01 QALYs per patient with suspected ischemic stroke. For each annual patient cohort, this translates to a total cost savings of US $11 million and a gain of 682 QALYs.	Favorable
3	Decision tree	Health care system and society	The incremental cost of ASPECTS^d^ interpretation with WhatsApp support compared to automated ASPECTS interpretation was US $220.94. The estimated treatment decision cost was US $618.28 over a 90-day period^e^.	Favorable
4	NA^f^	Health care system	Net cost savings from the AI-based management strategy of US $72 per screened patient in the first year after screening.	Favorable
5	NA	Health care system and society	The cost per screening for two CAD (computer-aided detection) software was lower than that of a radiologist (US $0.70-$0.93) for the high-yield scenarios studied. Projecting nationwide implementation, the additional cost of deploying CAD software was US $2.65 to US $19.3 million compared to US $23.97 million for human readers over a 4-year period.	Favorable
6	Not mentioned	Health care service provider	AI-assisted HIV and tuberculosis detection had an ICER of US $4620.47 per QALY, above Malawi’s cost-effectiveness threshold, making it cost-ineffective compared to conventional screening within the 56-day trial period.	Unfavorable
7	Decision tree	Health care service provider	Screening strategies using AI-based chest radiographs compared to initial smear microscopy and GeneXpert projected cost savings of US $23,233 and US $34,346 per 1000 people, respectively, and prevented 3%‐4% and 4% of DALYs^g^ over a 1-year time horizon.	Favorable
8	NA	NA	The algorithms showed underdiagnosis bias in three large public datasets based on gender, ethnicity or race, age, socioeconomic status, and intersectional subgroups.	Unfavorable
9	Markov	Society	At a willingness to pay of US $100,000 per QALY over a twenty-year analysis period, AI support for initial screening is cost-effective up to a cost of US $1240 per patient, and the ICER remains negative up to a threshold of US $68.	Favorable

a ICER: Incremental Cost-Effectiveness Ratio.

b QALY: Quality-Adjusted Life Years.

c AI: artificial intelligence.

d ASPECTS: Alberta stroke program early CT score.

e *: considering that £1 was equivalent to US $0.06349 on May 1, 2020 [36].

f NA: not applicable.

g DALY: disability-adjusted life years.

### Equity Evaluation

Only a single study focusing on equity evaluation was identified. This study used extensive datasets containing images from over 129,000 patients. The demographics of these patients displayed an almost equal distribution across genders, with the majority aged between 40 years and 80 years. The authors developed various AI models for detecting anomalies in chest x-rays. The findings from the equity evaluation indicated signs of underdiagnosis in AI-based interpretations of these imaging examinations, especially within specific subpopulations. For a comprehensive summary of the methodological details of this equity evaluation study, refer to Table 6, under study ID: 8.

## Discussion

### Principal Findings

This study presents a novel systematic review that examines the economic and equity implications of AI in medical diagnostic imaging across dermatology, neurology, and pulmonology. It stands out from previous research [9] by incorporating equity considerations and expanding the scope of medical specialties beyond those traditionally studied. The findings of this systematic review suggest that AI integration could provide economic benefits to health care systems, as the majority of included studies indicate that the technology is cost-effective. However, only 1 study assessed AI from an equity perspective, highlighting the issue of underdiagnosis, particularly among subgroups defined by gender, ethnicity, and socioeconomic status. This underscores the need for further research to ensure AI models are both cost-effective and equitable in clinical practice.

An important observation from this review is the notable scarcity of studies dedicated to exploring the economic and equity impacts of AI in the diagnostic imaging of dermatological, neurological, and pulmonary diseases. The bulk of the research has centered on pulmonology, tackling conditions like TB and lung cancer, followed by neurology. Dermatology emerged as the least studied field despite the availability of numerous open datasets for training models on skin lesions. The generalizability of AI algorithms and their practical application in clinical settings remains an unresolved issue. There is a necessity for real-life clinical trials using AI algorithms to broaden their applicability in routine medical practice. Dermatology, in particular, poses a unique challenge due to the diversity of skin tones, which requires a varied dataset for effective algorithm training [38].

Moreover, this review has also underscored a remarkable deficiency in studies investigating the aspect of equity in AI applications. This observation highlights the necessity for more inclusive research focused on equity-related issues. Furthermore, it is critical to acknowledge the potential influence of publication bias, where studies yielding positive outcomes are more likely to be published, thereby shaping the literature landscape [39].

A significant proportion of the studies included were conducted in high-income countries. Even studies involving low-income countries often collaborated with institutions from high-income countries. This trend raises important questions about the applicability of these findings in health care settings with limited resources. Some authors argue that the adoption of AI in low- and middle-income countries should follow a distinct path, rather than in high-income countries due to differences in staff, clinical experience, disease profiles, demographics, digital infrastructure, and available medical equipment. Health care facilities facing resource constraints require an integrated approach that encompasses clinical training, infrastructure enhancement, and a gradual introduction of AI technologies [40].

A key concern highlighted in this review is the insufficient detail provided about the AI tools used in the studies. Many articles did not disclose critical details such as the technology’s specific name, architectural framework, training and validation datasets, manufacturer, availability, and licensing details. These omissions limit result reproducibility and hinder health care professionals’ ability to implement AI technologies in clinical practice [41]. To address this gap, we emphasize the need for studies to follow established AI reporting guidelines, such as the MINIMAR (Minimum Information for Medical AI Reporting) framework, which advocates for transparency in AI model development and validation [42]. Furthermore, future studies should include standardized performance metrics, report biases in training data, and disclose the impact of image acquisition parameters, particularly in public health settings where data variability is significant. Establishing clear reporting standards and regulatory expectations will be crucial in ensuring AI models are both clinically applicable and equitably implemented.

On the other hand, the descriptions of the study populations often lacked essential demographic details, such as age, gender, and race, despite large sample sizes. This paucity of data on the study populations might impede the extrapolation of results across various demographic groups, especially when evaluating the impact of AI from an equity standpoint [41,43]. To address potential biases in AI, it is crucial for future studies to incorporate detailed demographic information and ensure training datasets are diverse across race, gender, socioeconomic status, and geographic regions. In addition, AI models should undergo fairness testing and be adjusted using bias-mitigation techniques, such as reweighting algorithms and adversarial debiasing. Policy makers should establish guidelines for transparent reporting of model performance across subpopulations and encourage collaborations between AI developers and public health institutions to ensure AI applications are equitable and contextually appropriate for diverse health care settings.

This review also identified a gap in the information regarding the timeframes during which the medical images were collected and analyzed. Understanding the timing of data collection and analysis is crucial for contextualizing the relevance of the findings. The dynamic nature of this field is reflected in the recent publication dates of the studies, indicating an evolving landscape where new technologies may emerge and existing ones may advance. Singh et al [44], 2021 reported a significant growth in the interest in medical applications of AI from 1998 to 2019, with a marked increase starting in 2015. Our systematic review, including articles regardless of publication date, found all relevant articles published post 2020, signifying a recent spike in scientific interest in evaluating AI technologies for potential health care integration.

The transferability of these technologies to Brazil’s public health system presented significant challenges. Only 2 of the studies were considered relevant to the Brazilian context, a finding that resonates with similar observations in the global research community. This highlights the potential for applying these findings to other countries with comparable demographics and health care circumstances. Numerous studies from various countries have also reported challenges in adapting AI technologies to their unique health care systems. This situation underscores the necessity for crafting tailored strategies to facilitate the effective international transfer of medical technologies. To make AI interventions more effective and globally relevant, it is necessary to promote region-specific research that accounts for demographic, socioeconomic, cultural, and infrastructure variations [41].

In dermatology, real-world clinical trials are particularly critical due to the diversity of skin tones, environmental exposures, and disease presentations across populations. Data collection should be standardized and consider cultural and socioeconomic variables that may impact model performance. This can be achieved using mixed-methods research designs, including qualitative interviews and focus groups to identify context-specific factors, followed by quantitative observational studies (eg, cross-sectional and longitudinal cohort studies) to assess data quality and variable distribution. Pilot studies and feasibility trials are also valuable to test recruitment strategies, data collection workflows, and model deployment logistics in diverse settings. Data analysis should involve appropriate statistical methods, such as stratified analyses and interaction testing, to validate the model’s effectiveness across different populations and clinical contexts. These methodological steps are essential for informing the design of robust, equitable clinical trials that evaluate AI-based interventions. In summary, future research should prioritize external validation of AI models on datasets that reflect social determinants of health to ensure equitable performance. In addition, partnerships between research institutions in low- and middle-income countries and international organizations could facilitate the adaptation and implementation of AI solutions, while also ensuring that developed models are more representative of the populations served. Incorporating standardized evaluation frameworks and transparency in AI reporting will further support the safe and effective deployment of dermatology-focused AI in diverse health care environments.

The only economic evaluation study reporting a disadvantageous outcome for AI use compared to standard care established a cost-effectiveness threshold of up to US $1200 per QALY [31]. This threshold, variable across nations, is influenced by factors like per capita income, public preferences, resource availability, and health policy, yet has inherent limitations. For example, in Brazil, the threshold is set at US $7914 per QALY (considering that US $1 was equivalent to BRL R$5.05 on October 20, 2023), increasing to US $23,744 per QALY for severe and rare diseases [45]. In the United Kingdom, the range is between US $24,335 and US $36,502 per QALY gained (considering that US $1 was equivalent to GBP 0.82 on October 20, 2023) [46]. To better contextualize AI cost-effectiveness, it is essential to examine cost thresholds across different health care systems. Given the economic disparities between high-, middle-, and low-income countries, AI adoption must be evaluated within country-specific willingness-to-pay thresholds. In low- to middle-income countries, where health care budgets are more constrained, AI solutions must demonstrate cost savings that justify their implementation, particularly when compared to existing diagnostic methods. Policy makers should consider not only the direct costs of AI but also its long-term benefits, such as improved diagnostic accuracy, reduced hospitalizations, and enhanced workforce efficiency. Future research should explore region-specific economic models to determine realistic AI pricing structures that optimize health care accessibility and sustainability.

The studies in this review imply AI’s potential to enhance cost-effectiveness, efficiency, and quality of life in health care, aligning with other research demonstrating AI as a cost-saving diagnostic tool. AI’s acceleration of diagnosis, reduction of subjectivity, and facilitation of treatment allow health care systems to allocate resources more efficiently, yielding tangible benefits and cost reductions. Hence, AI applications are emerging as an innovative strategy potentially beneficial to patient care, with prospects for widespread adoption and long-term cost minimization [47].

However, in terms of equity, the sole study meeting the criteria indicated unfavorable outcomes, highlighting underdiagnosis in specific subgroups. This is significant as health equity is a core principle in health care, and AI should not perpetuate or intensify existing disparities. Other studies have also shown that, despite accuracy in some AI models, racial disparities can persist [18]. To ensure equity, it is recommended that AI models incorporate indicators of social determinants, such as environmental, occupational, and income exposures. Efforts should be made to integrate the full etiological “context” of a patient’s health and optimize these models [43].

This study has limitations, including the potential bias of the chosen databases towards North American and European studies, possibly omitting relevant research from other regions, particularly Asia. Furthermore, the exclusion of gray literature, such as articles in open repositories like arXiv, might have constrained the scope of our review. Another significant challenge was the variability in study designs, data sources, and methodological approaches across the reviewed articles. This diversity made it difficult to synthesize the results comprehensively, rendering a meta-analysis unfeasible. Finally, the focus of this study on three specific medical fields—dermatology, neurology, and pulmonology—meant dealing with a limited number of available primary studies. This focus inherently excluded a vast array of studies in other medical areas, potentially overlooking relevant findings and insights that could be applicable to the broader discussion of AI in health care.

In summary, this systematic review emphasizes the imperative for a more robust and inclusive approach to using AI in medical imaging diagnostics, with particular attention to equity. Although the reviewed studies showcase AI’s potential in enhancing medical diagnostics, it’s crucial to ensure that such technologies do not deepen or perpetuate existing health disparities. Transparency in the depiction of AI tools and ensuring that study populations are representative are essential factors for consideration in future research endeavors. With AI rapidly integrating into health care, its comprehensive evaluation is necessary to guarantee that its benefits are accessible to all patients, irrespective of race, age, gender, or socioeconomic background. In addition, it is important to recognize that technologies developed within specific contexts, such as in high-income countries or for predominantly white populations, might not seamlessly transfer to other contexts or nations.

### Conclusion

This review underscores the importance of transparency in the description of AI tools and representativeness of population subgroups to mitigate health disparities. As AI is rapidly being integrated into health care, detailed assessments are essential to ensure that benefits reach all patients, regardless of sociodemographic factors.

## Supplementary material

10.2196/56240 Multimedia Appendix 1 Search strategy on economic evaluations or equity in the use of artificial intelligence tools for diagnostic support in imaging examinations in the dermatological area.

10.2196/56240 Multimedia Appendix 2 Search strategy on economic evaluations or equity in the use of artificial intelligence tools for diagnostic support in imaging examinations in the pulmonary area.

10.2196/56240 Multimedia Appendix 3 Search strategy on economic evaluations or equity in the use of artificial intelligence tools for diagnostic support in imaging examinations in the neurological area.

10.2196/56240 Multimedia Appendix 4 Search strategy on economic evaluations or equity in the use of artificial intelligence tools for diagnostic support in imaging examinations.

10.2196/56240 Multimedia Appendix 5 Summary of the articles selected for the study.

10.2196/56240 Checklist 1 PRISMA (Preferred Reporting Items for Systematic Reviews and Meta-Analyses) checklist.

## Abbreviations

AI: artificial intelligence
CHEC: Consensus on Health Economic Criteria
EPHPP: Effective Public Health Practice Project
ICER: incremental cost-effectiveness ratio
MINIMAR: Minimum Information for Medical AI Reporting
PICO: Population, Intervention, Comparison, and Outcome
PRISMA: Preferred Reporting Items for Systematic Reviews and Meta-Analyses
PRISMA-E: Preferred Reporting Items for Systematic Reviews and Meta-Analyses - Equity Extension
PROADI-SUS: Programa de Apoio ao Desenvolvimento Institucional do Sistema Único de Saúde
PROSPERO: International Prospective Register of Systematic Reviews
QALY: quality-adjusted life year
TB: tuberculosis
