# Supplementary material for: Economic Evaluations and Equity in the Use of Artificial Intelligence in Imaging Examinations for Medical Diagnosis in People With Dermatological, Neurological, and Pulmonary Diseases: Systematic Review
Source: Interact J Med Res. 2025 Aug 13;14:e56240. doi: 10.2196/56240 (PMC12349886; doi:10.2196/56240)
Supplement: Multimedia Appendix 1 [file ijmr-v14-e56240-s001.docx]

*Supplementary file 2:* Search strategy on economic evaluations or equity in the use of artificial intelligence tools for diagnostic support in imaging exams in dermatological area.

| Database | Search strategy |
| --- | --- |
| PubMed | ("Melanoma"[MeSH Terms] OR "Skin neoplasms"[MeSH Terms] OR "Neuroendocrine tumors"[MeSH Terms] OR "skin/pathology"[MeSH Terms] OR "Malignant melanoma*"[Text Word] OR "melanoma*"[Text Word] OR "Cutaneous melanoma"[Text Word] OR "Skin cancer*"[Text Word] OR "Skin lesion*"[Text Word] OR "Dermatopathology"[Text Word] OR "Dermatopathologie*"[Text Word] OR "Skin neoplasm*"[Text Word] OR "Skin infection*" [Text Word] OR "Skin infectious" [Text Word]) AND ("Cost of Illness"[MeSH Terms] OR "Cost-Benefit Analysis"[MeSH Terms] OR "Health Care Costs"[MeSH Terms] OR "models, economic"[MeSH Terms] OR "cost efficiency analysis"[Text Word] OR "cost-utility"[Text Word] OR "cost-effectiveness"[Text Word] OR "Cost of Illness" [Text Word] OR "Black People"[MeSH Terms] OR "Black or African American"[MeSH Terms] OR "White People"[MeSH Terms] OR "Vulnerable Populations"[MeSH Terms] OR "Race Factors"[MeSH Terms] OR "Poverty"[MeSH Terms] OR "Health Status Disparities"[MeSH Terms] OR "Racism"[MeSH Terms] OR "Prejudice"[MeSH Terms] OR "Socioeconomic factors"[MeSH Terms] OR "Health Status Disparities"[MeSH Terms] OR "Health Inequities"[MeSH Terms] OR "Social Determinants of Health"[MeSH Terms] OR "Equity"[Text Word] OR "Health equity"[Text Word]) AND ("Artificial Intelligence"[MeSH Terms] OR "Artificial Intelligence"[Text Word] OR "Artificial narrow intelligence"[Text Word] OR "Artificial General Intelligence"[Text Word] OR "Machine learning"[Text Word] OR "Deep Learning"[Text Word] OR "Neural Networks"[Text Word] OR "Algorithms"[Text Word] OR "Clinical Decision-Making"[MeSH Terms] OR "diagnosis, computer assisted"[MeSH Terms] OR "computer aided diagnosis"[Text Word] OR "Clinical decision support"[Text Word]) |
| Embase | ('melanoma'/exp OR 'skin tumor'/exp OR 'skin pathology'/exp) AND ('cost of illness'/exp OR 'cost benefit analysis'/exp OR 'health care cost'/exp OR 'economic model'/exp OR 'cost effectiveness analysis'/exp OR 'cost utility analysis'/exp OR 'black person'/exp OR 'african american'/exp OR 'african'/exp OR 'vulnerable population'/exp OR 'race'/exp OR 'poverty'/exp OR 'racism'/exp OR 'prejudice'/exp OR 'health disparity'/exp OR 'social determinants of health'/exp OR equity:ti,ab,kw OR 'health equity'/exp) AND ('machine learning'/exp OR 'learning algorithm'/exp OR 'deep learning'/exp OR 'feature learning (machine learning)'/exp OR 'neural network'/exp OR 'imaging algorithm'/exp OR 'clinical decision making'/exp OR 'clinical decision support system'/exp OR 'computer aided diagnosis'/exp OR 'artificial intelligence'/exp) |
| Scopus | ( TITLE-ABS-KEY ( "Melanoma" )  OR  TITLE-ABS-KEY ( "Skin neoplasms" )  OR  TITLE-ABS-KEY ( "Malignant melanoma" )  OR  TITLE-ABS-KEY ( "Cutaneous melanoma" )  OR  TITLE-ABS-KEY ( "Skin cancer" )  OR  TITLE-ABS-KEY ( "Skin lesion" )  OR  TITLE-ABS-KEY ( "Dermatopathology" ) )  AND  ( TITLE-ABS-KEY ( "Cost of Illness" )  OR  TITLE-ABS-KEY ( "Cost-Benefit Analysis" )  OR  TITLE-ABS-KEY ( "Health Care Costs" )  OR  TITLE-ABS-KEY ( "models, economic" )  OR  TITLE-ABS-KEY ( "cost efficiency analysis" )  OR  TITLE-ABS-KEY ( "cost-utility" )  OR  TITLE-ABS-KEY ( "cost-effectiveness" )  OR  TITLE-ABS-KEY ( "Black People" )  OR  TITLE-ABS-KEY ( "Black or African American" )  OR  TITLE-ABS-KEY ( "White People" )  OR  TITLE-ABS-KEY ( "Vulnerable Populations" )  OR  TITLE-ABS-KEY ( "Race Factors" )  OR  TITLE-ABS-KEY ( "Poverty" )  OR  TITLE-ABS-KEY ( "Health Status Disparities" )  OR  TITLE-ABS-KEY ( "Racism" )  OR  TITLE-ABS-KEY ( "Prejudice" )  OR  TITLE-ABS-KEY ( "Health Status Disparities" )  OR  TITLE-ABS-KEY ( "Health Inequities" )  OR  TITLE-ABS-KEY ( "Social Determinants of Health" )  OR  TITLE-ABS-KEY ( "Equity" )  OR  TITLE-ABS-KEY ( "Health equity" ) )  AND  ( TITLE-ABS-KEY ( "Artificial Intelligence" )  OR  TITLE-ABS-KEY ( "Artificial narrow intelligence" )  OR  TITLE-ABS-KEY ( "Artificial General Intelligence" )  OR  TITLE-ABS-KEY ( "Machine learning" )  OR  TITLE-ABS-KEY ( "Deep Learning" )  OR  TITLE-ABS-KEY ( "Neural Networks" )  OR  TITLE-ABS-KEY ( "Algorithms" )  OR  TITLE-ABS-KEY ( "Clinical Decision-Making" )  OR  TITLE-ABS-KEY ( "diagnosis, computer assisted" )  OR  TITLE-ABS-KEY ( "computer aided diagnosis" )  OR  TITLE-ABS-KEY ( "Clinical decision support" ) ) |
| Web of Science | (TS=("Melanoma" OR "Skin tumor" OR "Skin tumours" OR "Skin pathology")) AND (TS=(“cost of illness” OR “cost benefit analysis” OR “health care cost” OR “economic model” OR “cost effectiveness analysis” OR "cost effectiveness" OR “cost utility analysis” OR “black person*” OR “african american” OR “african” OR “vulnerable population” OR “race” OR “poverty” OR “racism” OR “prejudice” OR “health disparity” OR “social determinants of health” OR “equity” OR “health equity”)) AND (TS=(“machine learning” or “learning algorithm” or “deep learning” or “feature learning” or “neural network” or “imaging algorithm” or “clinical decision making” or “clinical decision support system” or “clinical decision support” or “computer aided diagnosis” or “artificial intelligence” or “Artificial narrow intelligence” or “Artificial General Intelligence” or “algorithm*”)) |
