# Supplementary material for: Economic Evaluations and Equity in the Use of Artificial Intelligence in Imaging Examinations for Medical Diagnosis in People With Dermatological, Neurological, and Pulmonary Diseases: Systematic Review
Source: Interact J Med Res. 2025 Aug 13;14:e56240. doi: 10.2196/56240 (PMC12349886; doi:10.2196/56240)
Supplement: Multimedia Appendix 3 [file ijmr-v14-e56240-s003.docx]

*Supplementary file 4:* Search strategy on economic evaluations or equity in the use of artificial intelligence tools for diagnostic support in imaging exams in neurological area

| Database | Search strategy |
| --- | --- |
| PubMed | ("Central Nervous System Diseases"[MeSH Terms] OR "Brain diseases"[MeSH Terms] OR "Brain Injuries"[MeSH Terms] OR "Intracranial Arterial Diseases"[MeSH Terms] OR "Cerebrovascular Disorders"[MeSH Terms] OR "Intracranial Aneurysm"[MeSH Terms] OR "Brain Neoplasms"[MeSH Terms] OR "Cerebral Ventricle Neoplasms"[MeSH Terms] OR "Glioma"[MeSH Terms] OR "Stroke"[MeSH Terms] OR "Hemorrhagic Stroke"[MeSH Terms] OR "Embolic Stroke"[MeSH Terms] OR "Thrombotic Stroke"[MeSH Terms] OR "Ischemic Stroke"[MeSH Terms] OR "Intracranial Hemorrhages"[MeSH Terms] OR "Multiple Sclerosis"[MeSH Terms] OR "Demyelinating Autoimmune Diseases, CNS"[MeSH Terms] OR "Dementia"[MeSH Terms] OR "Alzheimer Disease"[MeSH Terms] OR "Cognitive Dysfunction"[MeSH Terms] OR "Parkinson Disease"[MeSH Terms] OR "Brain injur*" [Text Word] OR "Parkinson Disease*" [Text Word] OR "Glioma*"[Text Word]) AND (("Neuroimaging"[MeSH Terms] OR "Functional Neuroimaging"[MeSH Terms] OR "imaging, three dimensional"[MeSH Terms] OR "image processing, computer assisted"[MeSH Terms] OR "tomography scanners, x ray computed"[MeSH Terms] OR "CT Scan"[Text Word] OR "Tomography" [Text Word] OR "Magnetic resonance imaging"[MeSH Terms] OR "Magnetic resonance"[Text Word] OR "MRI"[Text Word] OR "Functional MRI"[Text Word] OR "fMRI"[Text Word]) OR "Radiology"[MeSH Terms] OR "Diagnostic Imaging"[MeSH Terms] OR "Tomography, X-Ray Computed"[MeSH Terms] OR "Multiparametric Magnetic Resonance Imaging"[MeSH Terms] OR "Diffusion Magnetic Resonance Imaging"[MeSH Terms]) AND ("Cost of Illness"[MeSH Terms] OR "Cost-Benefit Analysis"[MeSH Terms] OR "Health Care Costs"[MeSH Terms] OR "models, economic"[MeSH Terms] OR "cost efficiency analysis"[Text Word] OR "cost-utility"[Text Word] OR "cost-effectiveness"[Text Word] OR "Black People"[MeSH Terms] OR "Black or African American"[MeSH Terms] OR "White People"[MeSH Terms] OR "Vulnerable Populations"[MeSH Terms] OR "Race Factors"[MeSH Terms] OR "Poverty"[MeSH Terms] OR "Health Status Disparities"[MeSH Terms] OR "Racism"[MeSH Terms] OR "Prejudice"[MeSH Terms] OR "Socioeconomic factors"[MeSH Terms] OR "Health Status Disparities"[MeSH Terms] OR "Health Inequities"[MeSH Terms] OR "Social Determinants of Health"[MeSH Terms] OR "Equity"[Text Word] OR "Health equity"[Text Word]) AND ("Artificial Intelligence"[MeSH Terms] OR "Artificial Intelligence"[Text Word] OR "Artificial narrow intelligence"[Text Word] OR "Artificial General Intelligence"[Text Word] OR "Machine learning"[Text Word] OR "Deep Learning"[Text Word] OR "Neural Networks"[Text Word] OR "Algorithms"[Text Word] OR "Clinical Decision-Making"[MeSH Terms] OR "diagnosis, computer assisted"[MeSH Terms] OR "computer aided diagnosis"[Text Word] OR "Clinical decision support"[Text Word]) |
| Embase | ('brain disease'/exp OR 'brain disease' OR 'cerebral artery disease'/exp OR 'cerebral artery disease' OR 'cerebrovascular disease'/exp OR 'cerebrovascular disease' OR 'intracranial aneurysm'/exp OR 'intracranial aneurysm' OR 'brain tumor'/exp OR 'brain tumor' OR 'glioma'/exp OR 'glioma' OR 'cerebrovascular accident'/exp OR 'cerebrovascular accident' OR 'cardioembolic stroke'/exp OR 'cardioembolic stroke' OR 'ischemic stroke'/exp OR 'ischemic stroke' OR 'brain hemorrhage'/exp OR 'brain hemorrhage' OR 'multiple sclerosis'/exp OR 'multiple sclerosis' OR 'demyelinating disease'/exp OR 'demyelinating disease' OR 'dementia'/exp OR 'dementia' OR 'cognitive defect'/exp OR 'cognitive defect' OR 'parkinson disease'/exp OR 'parkinson disease') AND ('neuroimaging'/exp OR 'neuroimaging' OR 'functional neuroimaging'/exp OR 'functional neuroimaging' OR 'three-dimensional imaging'/exp OR 'three-dimensional imaging' OR 'image processing'/exp OR 'image processing' OR 'ct scanner'/exp OR 'ct scanner' OR 'x-ray computed tomography'/exp OR 'x-ray computed tomography' OR 'nuclear magnetic resonance imaging'/exp OR 'nuclear magnetic resonance imaging' OR 'radiodiagnosis'/exp OR 'radiodiagnosis' OR 'diagnostic imaging'/exp OR 'diagnostic imaging' OR 'multiparametric magnetic resonance imaging'/exp OR 'multiparametric magnetic resonance imaging' OR 'diffusion weighted imaging'/exp OR 'diffusion weighted imaging' OR 'magnetic resonance':ti,ab,kw OR 'mri':ti,ab,kw OR 'functional mri':ti,ab,kw OR 'fmri':ti,ab,kw OR 'tomography':ti,ab,kw) AND ('cost of illness'/exp OR 'cost of illness' OR 'cost benefit analysis'/exp OR 'cost benefit analysis' OR 'health care cost'/exp OR 'health care cost' OR 'economic model'/exp OR 'economic model' OR 'cost effectiveness analysis'/exp OR 'cost effectiveness analysis' OR 'cost utility analysis'/exp OR 'cost utility analysis' OR 'black person'/exp OR 'black person' OR 'african american'/exp OR 'african american' OR 'african'/exp OR 'african' OR 'vulnerable population'/exp OR 'vulnerable population' OR 'race'/exp OR 'race' OR 'poverty'/exp OR 'poverty' OR 'racism'/exp OR 'racism' OR 'prejudice'/exp OR 'prejudice' OR 'health disparity'/exp OR 'health disparity' OR 'social determinants of health'/exp OR 'social determinants of health' OR equity:ti,ab,kw OR 'health equity'/exp OR 'health equity') AND ('machine learning'/exp OR 'machine learning' OR 'learning algorithm'/exp OR 'learning algorithm' OR 'deep learning'/exp OR 'deep learning' OR 'feature learning (machine learning)'/exp OR 'feature learning (machine learning)' OR 'neural network'/exp OR 'neural network' OR 'imaging algorithm'/exp OR 'imaging algorithm' OR 'clinical decision making'/exp OR 'clinical decision making' OR 'clinical decision support system'/exp OR 'clinical decision support system' OR 'computer aided diagnosis'/exp OR 'computer aided diagnosis' OR 'artificial intelligence'/exp OR 'artificial intelligence') |
| Scopus | ( TITLE-ABS-KEY ( "Central Nervous System Disease*" )  OR  TITLE-ABS-KEY ( "Brain disease*" )  OR  TITLE-ABS-KEY ( "Brain Injur*" )  OR  TITLE-ABS-KEY ( "Intracranial Arterial Disease*" )  OR  TITLE-ABS-KEY ( "Cerebrovascular Disorder*" )  OR  TITLE-ABS-KEY ( "Intracranial Aneurysm*" )  OR  TITLE-ABS-KEY ( "Brain Neoplasm*" )  OR  TITLE-ABS-KEY ( "Cerebral Ventricle Neoplasm*" )  OR  TITLE-ABS-KEY ( "Glioma*" )  OR  TITLE-ABS-KEY ( "Stroke" )  OR  TITLE-ABS-KEY ( "Hemorrhagic Stroke" )  OR  TITLE-ABS-KEY ( "Embolic Stroke" )  OR  TITLE-ABS-KEY ( "Thrombotic Stroke" )  OR  TITLE-ABS-KEY ( "Ischemic Stroke" )  OR  TITLE-ABS-KEY ( "Intracranial Hemorrhag*" )  OR  TITLE-ABS-KEY ( "Multiple Sclerosis" )  OR  TITLE-ABS-KEY ( "Demyelinating Autoimmune Diseases" )  OR  TITLE-ABS-KEY ( "Dementia" )  OR  TITLE-ABS-KEY ( "Alzheimer Disease*" )  OR  TITLE-ABS-KEY ( "Cognitive Dysfunction" )  OR  TITLE-ABS-KEY ( "Parkinson Disease*" ) )  AND  ( TITLE-ABS-KEY ( "Neuroimaging" )  OR  TITLE-ABS-KEY ( "Functional Neuroimaging" )  OR  TITLE-ABS-KEY ( "imaging, three dimensional" )  OR  TITLE-ABS-KEY ( "image processing, computer assisted" )  OR  TITLE-ABS-KEY ( "tomography scanners, x ray computed" )  OR  TITLE-ABS-KEY ( "CT Scan" )  OR  TITLE-ABS-KEY ( "Tomography" )  OR  ( "Magnetic resonance imaging" )  OR  TITLE-ABS-KEY ( "Magnetic resonance" )  OR  TITLE-ABS-KEY ( "MRI" )  OR  TITLE-ABS-KEY ( "Functional MRI" )  OR  TITLE-ABS-KEY ( "MRI" )  OR  TITLE-ABS-KEY ( "Radiodiagnosis" )  OR  TITLE-ABS-KEY ( "Diagnostic Imaging" )  OR  TITLE-ABS-KEY ( "Tomography, X-Ray Computed" )  OR  TITLE-ABS-KEY ( "Multiparametric Magnetic Resonance Imaging" )  OR  TITLE-ABS-KEY ( "Diffusion Magnetic Resonance Imaging" ) )  AND  ( TITLE-ABS-KEY ( "Cost of Illness" )  OR  TITLE-ABS-KEY ( "Cost-Benefit Analysis" )  OR  TITLE-ABS-KEY ( "Health Care Costs" )  OR  TITLE-ABS-KEY ( "models, economic" )  OR  TITLE-ABS-KEY ( "cost efficiency analysis" )  OR  TITLE-ABS-KEY ( "cost-utility" )  OR  TITLE-ABS-KEY ( "cost-effectiveness" )  OR  TITLE-ABS-KEY ( "Black People" )  OR  TITLE-ABS-KEY ( "Black or African American" )  OR  TITLE-ABS-KEY ( "White People" )  OR  TITLE-ABS-KEY ( "Vulnerable Populations" )  OR  TITLE-ABS-KEY ( "Race Factors" )  OR  TITLE-ABS-KEY ( "Poverty" )  OR  TITLE-ABS-KEY ( "Health Status Disparities" )  OR  TITLE-ABS-KEY ( "Racism" )  OR  TITLE-ABS-KEY ( "Prejudice" )  OR  TITLE-ABS-KEY ( "Health Status Disparities" )  OR  TITLE-ABS-KEY ( "Health Inequities" )  OR  TITLE-ABS-KEY ( "Social Determinants of Health" )  OR  TITLE-ABS-KEY ( "Equity" )  OR  TITLE-ABS-KEY ( "Health equity" ) )  AND  ( TITLE-ABS-KEY ( "Artificial Intelligence" )  OR  TITLE-ABS-KEY ( "Artificial narrow intelligence" )  OR  TITLE-ABS-KEY ( "Artificial General Intelligence" )  OR  TITLE-ABS-KEY ( "Machine learning" )  OR  TITLE-ABS-KEY ( "Deep Learning" )  OR  TITLE-ABS-KEY ( "Neural Networks" )  OR  TITLE-ABS-KEY ( "Algorithms" )  OR  TITLE-ABS-KEY ( "Clinical Decision-Making" )  OR  TITLE-ABS-KEY ( "diagnosis, computer assisted" )  OR  TITLE-ABS-KEY ( "computer aided diagnosis" )  OR  TITLE-ABS-KEY ( "Clinical decision support" ) ) |
| Web of Science | (TS=(“Central Nervous System Diseases” OR“Brain diseases” OR “Brain Injur*” OR “Intracranial Arterial Disease*” OR “Cerebrovascular Disorder*” OR “Intracranial Aneurysm” OR “Brain Neoplasm*” OR “Cerebral Ventricle Neoplasm*” OR “Glioma*” OR “Stroke” OR “Hemorrhagic Stroke” OR “Embolic Stroke” OR “Thrombotic Stroke” OR “Ischemic Stroke” OR “Intracranial Hemorrhage*” OR “Multiple Sclerosis” OR “Demyelinating Autmune Diseases, CNS” OR “Dementia” OR “Alzheimer Disease” OR “Cognitive Dysfunction” OR “Parkinson Disease”)) AND (TS=(“Neuroimaging" OR "Functional Neuroimaging" OR "Imaging, three dimensional" OR "image processing, computer assisted" OR "tomography scanners, x ray computed" OR “Tomography” OR "CT Scan" OR "Magnetic resonance imaging" OR "Magnetic resonance" OR "MRI" OR "Functional MRI" OR "fMRI" OR “Radiodiagnosis” OR “Diagnostic Imaging” OR “Tomography, X-Ray Computed” OR “Multiparametric Magnetic Resonance Imaging” OR “Diffusion Magnetic Resonance Imaging”)) AND (TS=(“cost of illness” OR “cost benefit analysis” OR “health care cost” OR “economic model” OR “cost effectiveness analysis” OR "cost effectiveness" OR “cost utility analys” OR “black person*” OR “african american” OR “african” OR “vulnerable population” OR “race” OR “poverty” OR “racism” OR “prejudice” OR “health disparity” OR “social determinants of health” OR “equity” OR “health equity”)) AND (TS=(“machine learning” or “learning algorithm” or “deep learning” or “feature learning” or “neural network” or “imaging algorithm” or “clinical decision making” or “clinical decision support system” or “clinical decision support” or “computer aided diagnosis” or “artificial intelligence” or “Artificial narrow intelligence” or “Artificial General Intelligence” or “algorithm*”)) |
