# Supplementary material for: Economic Evaluations and Equity in the Use of Artificial Intelligence in Imaging Examinations for Medical Diagnosis in People With Dermatological, Neurological, and Pulmonary Diseases: Systematic Review
Source: Interact J Med Res. 2025 Aug 13;14:e56240. doi: 10.2196/56240 (PMC12349886; doi:10.2196/56240)
Supplement: Multimedia Appendix 4 [file ijmr-v14-e56240-s004.docx]

*Supplementary file 5:* Search strategy on economic evaluations or equity in the use of artificial intelligence tools for diagnostic support in imaging exams

| Database | Search strategy |
| --- | --- |
| PubMed | ("Neuroimaging"[MeSH Terms] OR "Functional Neuroimaging"[MeSH Terms] OR "Diagnostic Imaging"[MeSH Terms] OR "Diagnostic imaging"[Text Word] OR "tomography, x ray computed"[MeSH Terms] OR "tomography scanners, x ray computed"[MeSH Terms] OR "Computed tomography"[Text Word] OR "Tomography scanner"[Text word] OR "Tomography" [Text word] OR "Radiography" [MESH Terms] OR "Radiography, thoracic"[MESH Terms] OR "Mass Chest X-Ray" [MESH Terms] OR "X-Ray" [Text word] OR "Thoracic radiography"[Text Word] OR "CT Scan"[Text Word] OR "Magnetic resonance imaging"[MeSH Terms] OR "Magnetic resonance"[Text Word] OR "MRI"[Text Word] OR "Functional MRI"[Text Word] OR "fMRI"[Text Word] OR "Radiology"[MeSH Terms] OR "Diagnostic Imaging"[MeSH Terms] OR "Multiparametric Magnetic Resonance Imaging"[MeSH Terms] OR "Diffusion Magnetic Resonance Imaging"[MeSH Terms] OR "imaging, three dimensional"[MeSH Terms] OR "image processing, computer assisted"[MeSH Terms]) AND ("Cost of Illness"[MeSH Terms] OR "Cost-Benefit Analysis"[MeSH Terms] OR "Health Care Costs"[MeSH Terms] OR "models, economic"[MeSH Terms] OR "cost efficiency analysis"[Text Word] OR "cost-utility"[Text Word] OR "cost-effectiveness"[Text Word] OR "Black People"[MeSH Terms] OR "Black or African American"[MeSH Terms] OR "White People"[MeSH Terms] OR "Vulnerable Populations"[MeSH Terms] OR "Race Factors"[MeSH Terms] OR "Poverty"[MeSH Terms] OR "Health Status Disparities"[MeSH Terms] OR "Racism"[MeSH Terms] OR "Prejudice"[MeSH Terms] OR "Socioeconomic factors"[MeSH Terms] OR "Health Status Disparities"[MeSH Terms] OR "Health Inequities"[MeSH Terms] OR "Social Determinants of Health"[MeSH Terms] OR "Equity"[Text Word] OR "Health equity"[Text Word]) AND ("Artificial Intelligence"[MeSH Terms] OR "Artificial Intelligence"[Text Word] OR "Artificial narrow intelligence"[Text Word] OR "Artificial General Intelligence"[Text Word] OR "Machine learning"[Text Word] OR "Deep Learning"[Text Word] OR "Neural Networks"[Text Word] OR "Algorithms"[Text Word] OR "Clinical Decision-Making"[MeSH Terms] OR "diagnosis, computer assisted"[MeSH Terms] OR "computer aided diagnosis"[Text Word] OR "Clinical decision support"[Text Word]) |
| Embase | ('neuroimaging'/exp OR 'functional neuroimaging'/exp OR 'three-dimensional imaging'/exp OR 'image processing'/exp OR 'ct scanner'/exp OR 'x-ray computed tomography'/exp OR 'nuclear magnetic resonance imaging'/exp OR 'diagnostic imaging'/exp OR 'multiparametric magnetic resonance imaging'/exp OR 'diffusion weighted imaging'/exp OR 'magnetic resonance':ti,ab,kw OR 'mri':ti,ab,kw OR 'functional mri':ti,ab,kw OR 'fmri':ti,ab,kw OR 'diagnostic imaging':ti,ab,kw OR 'x-ray tomography':ti,ab,kw OR 'ct scanner':ti,ab,kw OR 'computer assisted tomography':ti,ab,kw OR 'tomography':ti,ab,kw OR radiography:ti,ab,kw OR 'thorax radiography':ti,ab,kw OR 'mass chest':ti,ab,kw OR radiodiagnosis:ti,ab,kw OR 'radiodiagnosis'/exp OR 'radiodiagnosis') AND ('cost of illness'/exp OR 'cost benefit analysis'/exp OR 'health care cost'/exp OR 'economic model'/exp OR 'cost effectiveness analysis'/exp OR 'cost utility analysis'/exp OR 'black person'/exp OR 'african american'/exp OR 'african'/exp OR 'vulnerable population'/exp OR 'race'/exp OR 'poverty'/exp OR 'racism'/exp OR 'prejudice'/exp OR 'health disparity'/exp OR 'social determinants of health'/exp OR 'equity':ti,ab,kw OR 'health equity'/exp) AND ('machine learning'/exp OR 'learning algorithm'/exp OR 'deep learning'/exp OR 'feature learning (machine learning)'/exp OR 'neural network'/exp OR 'imaging algorithm'/exp OR 'clinical decision making'/exp OR 'clinical decision support system'/exp OR 'computer aided diagnosis'/exp OR 'artificial intelligence'/exp) |
| Scopus | ( TITLE-ABS-KEY ( "Neuroimaging" )  OR  TITLE-ABS-KEY ( "Functional Neuroimaging" )  OR  TITLE-ABS-KEY ( "imaging, three dimensional" )  OR  TITLE-ABS-KEY ( "image processing, computer assisted" )  OR  TITLE-ABS-KEY ( "tomography scanners, x ray computed" )  OR  TITLE-ABS-KEY ( "CT Scan" )  OR  TITLE-ABS-KEY ( "Tomography" )  OR  ( "Magnetic resonance imaging" )  OR  TITLE-ABS-KEY ( "Magnetic resonance" )  OR  TITLE-ABS-KEY ( "MRI" )  OR  TITLE-ABS-KEY ( "Functional MRI" )  OR  TITLE-ABS-KEY ( "MRI" )  OR  TITLE-ABS-KEY ( "Radiodiagnosis" )  OR  TITLE-ABS-KEY ( "Diagnostic Imaging" )  OR  TITLE-ABS-KEY ( "Tomography, X-Ray Computed" )  OR  TITLE-ABS-KEY ( "Multiparametric Magnetic Resonance Imaging" )  OR  TITLE-ABS-KEY ( "Diffusion Magnetic Resonance Imaging" )  OR  TITLE-ABS-KEY ( "diagnostic imaging" )  OR  TITLE-ABS-KEY ( "x-ray tomography" )  OR  TITLE-ABS-KEY ( "CT scanner" )  OR  TITLE-ABS-KEY ( "computer assisted tomography" )  OR  TITLE-ABS-KEY ( "radiography" )  OR  TITLE-ABS-KEY ( "thorax radiography" )  OR  TITLE-ABS-KEY ( "mass chest" )  OR  TITLE-ABS-KEY ( "x-ray diagnosis" )  OR  TITLE-ABS-KEY ( "Thoracic radiography" )  OR  TITLE-ABS-KEY ( "Mass chest x-ray" ) )  AND  ( TITLE-ABS-KEY ( "Cost of Illness" )  OR  TITLE-ABS-KEY ( "Cost-Benefit Analysis" )  OR  TITLE-ABS-KEY ( "Health Care Costs" )  OR  TITLE-ABS-KEY ( "models, economic" )  OR  TITLE-ABS-KEY ( "cost efficiency analysis" )  OR  TITLE-ABS-KEY ( "cost-utility" )  OR  TITLE-ABS-KEY ( "cost-effectiveness" )  OR  TITLE-ABS-KEY ( "Black People" )  OR  TITLE-ABS-KEY ( "Black or African American" )  OR  TITLE-ABS-KEY ( "White People" )  OR  TITLE-ABS-KEY ( "Vulnerable Populations" )  OR  TITLE-ABS-KEY ( "Race Factors" )  OR  TITLE-ABS-KEY ( "Poverty" )  OR  TITLE-ABS-KEY ( "Health Status Disparities" )  OR  TITLE-ABS-KEY ( "Racism" )  OR  TITLE-ABS-KEY ( "Prejudice" )  OR  TITLE-ABS-KEY ( "Health Status Disparities" )  OR  TITLE-ABS-KEY ( "Health Inequities" )  OR  TITLE-ABS-KEY ( "Social Determinants of Health" )  OR  TITLE-ABS-KEY ( "Equity" )  OR  TITLE-ABS-KEY ( "Health equity" ) )  AND  ( TITLE-ABS-KEY ( "Artificial Intelligence" )  OR  TITLE-ABS-KEY ( "Artificial narrow intelligence" )  OR  TITLE-ABS-KEY ( "Artificial General Intelligence" )  OR  TITLE-ABS-KEY ( "Machine learning" )  OR  TITLE-ABS-KEY ( "Deep Learning" )  OR  TITLE-ABS-KEY ( "Neural Networks" )  OR  TITLE-ABS-KEY ( "Algorithms" )  OR  TITLE-ABS-KEY ( "Clinical Decision-Making" )  OR  TITLE-ABS-KEY ( "diagnosis, computer assisted" )  OR  TITLE-ABS-KEY ( "computer aided diagnosis" )  OR  TITLE-ABS-KEY ( "Clinical decision support" ) ) |
| Web of Science | (TS=(“Neuroimaging" OR "Functional Neuroimaging" OR "Imaging, three dimensional" OR "image processing, computer assisted" OR "tomography scanners, x ray computed" OR “Tomography” OR "CT Scan" OR "Magnetic resonance imaging" OR "Magnetic resonance" OR "MRI" OR "Functional MRI" OR "fMRI" OR “Radiodiagnosis” OR “Diagnostic Imaging” OR “Tomography, X-Ray Computed” OR “Multiparametric Magnetic Resonance Imaging” OR “Diffusion Magnetic Resonance Imaging” OR "x-ray tomography" or "computed tomography" or "CT scanner" or "computer assited tomography" or "radiography" or "Thora* radiograh*" or "mass chest" or "X-ray diagnosis")) AND (TS=(“cost of illness” OR “cost benefit analysis” OR “health care cost” OR “economic model” OR “cost effectiveness analysis” OR "cost effectiveness" OR “cost utility analys” OR “black person*” OR “african american” OR “african” OR “vulnerable population” OR “race” OR “poverty” OR “racism” OR “prejudice” OR “health disparity” OR “social determinants of health” OR “equity” OR “health equity”)) AND (TS=(“machine learning” or “learning algorithm” or “deep learning” or “feature learning” or “neural network” or “imaging algorithm” or “clinical decision making” or “clinical decision support system” or “clinical decision support” or “computer aided diagnosis” or “artificial intelligence” or “Artificial narrow intelligence” or “Artificial General Intelligence” or “algorithm*”)) |
