# Supplementary material for: Economic Evaluations and Equity in the Use of Artificial Intelligence in Imaging Examinations for Medical Diagnosis in People With Dermatological, Neurological, and Pulmonary Diseases: Systematic Review
Source: Interact J Med Res. 2025 Aug 13;14:e56240. doi: 10.2196/56240 (PMC12349886; doi:10.2196/56240)
Supplement: Multimedia Appendix 5 [file ijmr-v14-e56240-s005.docx]

Supplementary 6: summary of the articles selected for the study.

| **ID** | **Summary** |
| --- | --- |
| 1 | Use of AI in skin photographs, dental caries radiography, and retinal images compared to standard procedures in the detection of melanoma, caries, and diabetes |
| 2 | Use of AI in CT angiography and standard procedures in the detection of stroke due to large intracranial vessel occlusions |
| 3 | Use of AI for automated scoring in ischemic stroke CT screening patients versus neuroradiologist analysis from CT images sent via messaging app |
| 4 | Diagnosis and follow-up of lung cancer based on isolated Lung-RADS lung CT screening reports and using an AI malignancy-risk score algorithm |
| 5 | Detection or screening of TB through chest radiography by radiologists and the use of commercially available computer-aided detection (CAD) software |
| 6 | Clinical trial to assess the effectiveness of HIV and TB diagnosis by AI-assisted chest radiography in relation to treatment time and disease prevalence |
| 7 | Use of AI-assisted chest radiography for screening patients with TB suggestive symptoms compared to standard procedure |
| 8 | Presence of bias in diagnosis by pathology search algorithms in chest radiographs by gender, age, ethnicity/race, and socioeconomic status |
| 9 | Use of AI-based chest CT for lung cancer screening compared to isolated chest CT |

AI: artificial intelligence; CT: computed tomography; TB: tuberculosis.

Computer-aided detection (CAD) refers to the use of software algorithms to assist healthcare professionals in identifying abnormalities in medical images, such as X-rays or CT scans.
